# SimQ: Real-Time Retrieval of Similar Consumer Health Questions

**DOI:** 10.2196/jmir.3388

**Published:** 2015-02-17

**Authors:** Jake Luo, Guo-Qiang Zhang, Susan Wentz, Licong Cui, Rong Xu

**Affiliations:** ^1^Center for Biomedical Data and Language ProcesssingDepartment of Health Informatics and AdministrationUniversity of Wisconsin MilwaukeeMilwaukee, WIUnited States; ^2^Division of Medical Informatics, Center for Clinical InvestigationSchool of MedicineCase Western Reserve UniversityCleveland, OHUnited States; ^3^Netwellness.orgSchool of MedicineCase Western Reserve UniversityCleveland, OHUnited States

**Keywords:** Online Health Information Seeking, Health Information Delivery, Consumer Health Informatics, Consumer Question Retrieval, Similarity Analysis, Netwellness.org, Health Care Questions, Search and Query

## Abstract

**Background:**

There has been a significant increase in the popularity of Web-based question-and-answer (Q&A) services that provide health care information for consumers. Large amounts of Q&As have been archived in these online communities, which form a valuable knowledge base for consumers who seek answers to their health care concerns. However, due to consumers’ possible lack of professional knowledge, it is still very challenging for them to find Q&As that are closely relevant to their own health problems. Consumers often repeatedly ask similar questions that have already been answered previously by other users.

**Objective:**

In this study, we aim to develop efficient informatics methods that can retrieve similar Web-based consumer health questions using syntactic and semantic analysis.

**Methods:**

We propose the “SimQ” to achieve this objective. SimQ is an informatics framework that compares the similarity of archived health questions and retrieves answers to satisfy consumers’ information needs. Statistical syntactic parsing was used to analyze each question’s syntactic structure. Standardized Unified Medical Language System (UMLS) was employed to annotate semantic types and extract medical concepts. Finally, the similarity between sentences was calculated using both semantic and syntactic features.

**Results:**

We used 2000 randomly selected consumer questions to evaluate the system’s performance. The results show that SimQ reached the highest precision of 72.2%, recall of 78.0%, and F-score of 75.0% when using compositional feature representations.

**Conclusions:**

We demonstrated that SimQ complements the existing Q&A services of Netwellness, a not-for-profit community-based consumer health information service that consists of nearly 70,000 Q&As and serves over 3 million users each year. SimQ not only reduces response delay by instantly providing closely related questions and answers, but also helps consumers to improve the understanding of their health concerns.

## Introduction

 Web-based health-related question and answering (Q&A) services are becoming more and more popular. Some consumer health websites receive millions of page views each year, such as NetWellness, WebMed, and EverydayHealth. Thousands of users visit these websites to search for answers related to their health problems [1]. Many of these health information websites are community-based, which means a user can submit a question to public forums and wait for that question to be answered by other users or experts.

The service model of community-based Q&A platforms has several unique advantages. First, users keep their identity anonymous, which protects the users’ privacy and encourages information sharing. For example, many people who feel too stressed or embarrassed to ask certain types of questions during face-to-face physician consultations (eg, sex-related issues, weight-related concerns, or emotional problems) can seek help from the online community. Second, the Q&A platform can serve as an information source for acquiring new knowledge. It enhances a user’s understanding of health care on many different topics, such as nutrition, patient care, or disease management. Third, compared to face-to-face physician consultation, a community-based service normally provides a quicker response and a wider range of perspectives. For example, a user who asks a question about “children nutrition” may receive answers from both child-care experts and nutritionists. Finally, the online community provides a platform for consumers to share their health concerns and wellness interests. This creates an environment that not only shares new knowledge, but also provides emotional support for health care consumers. Therefore, a community based Q&A is an excellent way of delivering health care information to a wide range of public users. It could help reduce the time and cost of information delivery, such as those services provided by MIMIR [2] and Yahoo Answers [3].

Despite the fact that an online community-based health information service has many advantages, there are still many challenging problems that need to be addressed to improve the service’s quality and accessibility [4]. Consumers are often unaware of the great value of archived historical questions. Also, many consumers may lack professional knowledge, making it challenging for them to find Q&As that are relevant to their own particular health concerns. Often times, these users post similar questions that have been answered previously. As a result, duplicate questions delay service responses and create additional burdens for the service platform, which then becomes a significant waste of valuable resources. Furthermore, domain experts and administrators also have a strong need to retrieve and group similar Q&As to support content management. To address these problems, a similarity-based Q&A retrieval system is highly desirable both for health consumers and domain administrators to accommodate their specific needs.

Many community-based service platforms have now archived thousands of Q&As, which creates a valuable knowledge base. Berland et al published a study [4] on evaluating consumer health platforms on the Internet. The results show that the retrieval of relevant information is a critical factor for effectively delivering health information to consumers. Developing efficient methods to retrieve similar questions on the Q&A platforms can help unleash the power of the archived Q&A as important knowledge bases, and make the archived information more accessible to consumers. In this paper, the SimQ project is proposed as a useful framework that focuses on developing new methods to retrieve similar questions from the large health information platform, NetWellness [5,6].

NetWellness is a not-for-profit health information website, which has been providing consistent and high quality services for consumers since 1995. This service platform is operated by professional health care experts from three universities, including Case Western Reserve University, Ohio State University, and the University of Cincinnati. The health information provided by the NetWellness website has been evaluated and maintained at high quality standards by experts who periodically review the content to ensure that the information is up-to-date. Over 500 health experts, including physicians, nurses, pharmacists, dieticians, dentists, genetics counselors, optometrists, athletic trainers, and social workers have contributed to the public Q&A, and more importantly, provide professional health care information that directly addresses consumers’ health concerns. Over 70,000 consumer questions have been answered with approximately 1,400,000 [7] visits reaching the website each month. NetWellness continuously collects user feedback through Web-based surveys. Close to 80% (28,137/35,719) of the users said that NetWellness Q&As were very useful for them but, surprisingly, about 67% (17,647/26,257) of users reported that the health information they found on the site was “new” to them. Similar to Lau and Coiera’s report [8], the survey clearly indicates a strong need for developing advanced informatics tools to provide more informative and relevant knowledge to educate users and to fulfill consumers’ health information needs. The goal of the present paper is to develop a *semantic similarity analysis* method to support the need for retrieving similar questions from NetWellness, that complements existing services, and that enables efficient reuse of the accumulated Q&A knowledge (source code available in Multimedia Appendix 1).

Similarity analysis of Q&As remains a challenging task [9]. There are several related studies that aim to develop new methods to improve the Q&A systems in the information retrieval research field. Metzler and Croft [10] presented a support vector machine (SVM) based question classification method, in which the trained classifier facilitates the determination of fact-based question types, such as the question, “What is the world’s highest peak?”, which can be classified to “location” question types. Sneiders [11] proposed a method that uses question templates to transfer questions into database queries, which query the answers based on the predefined variable slots into the templates. This method provides a formal way to construct a database query from structured question variables. However, due to the requirement of laborious effort for developing templates for each type of question, that method is not scalable for large and open question databases. More recently, a ranking framework [12] was proposed to retrieve relevant content from social media by using community feedback, such as the user’s experience, reputation, and vote. This method is typically effective when the community allows users to evaluate the questions openly and provide feedback. Wang et al [13] proposed a method that uses syntactic structure to find similar questions. This method was tested on Yahoo Answers, which showed that the use of syntactic structure performed better than the traditional “bag-of-words” feature representation. Cui et al [6,14] recently proposed another method that uses multi-topic navigation to help consumers navigate question archives.

These methods provide different solutions to improve Q&A retrieval on various domains, such as question classification and ranking. However, health care Q&As often contain challenging medical information that are too difficult to encapsulate for standard language processing and information retrieval to be effective [14-16] (eg, description of diseases, signs and symptoms, pharmacological reactions, etc). In this paper, we propose a different method that takes advantage of the semantic network of the Unified Medical Language System (UMLS) [17] to assign semantic annotations to consumer health questions. The semantic features combined with statistical syntactic parsing results are then used to calculate similarity scores and retrieve similar questions. The goal is to provide similar Q&As that can help consumers better understand their own health concerns.

## Methods

### Challenge

**Figure 1:**
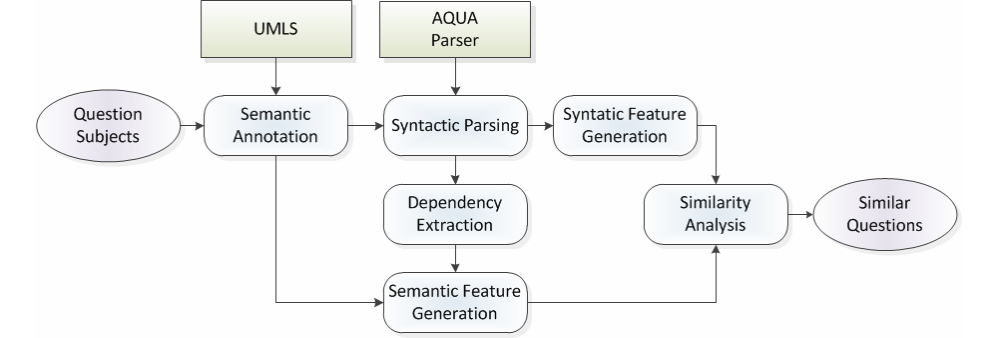
Overview of the SimQ framework for analyzing similarity of consumer health question.

Questions submitted to the NetWellness website are written in free-text, which contains complex syntactic structure and semantic elements. Analyzing the similarity of consumer questions is not a simple task, so we propose a method that combines semantic annotation techniques and syntactic natural language processing methods to analyze the question similarity. Figure 1 shows the overall framework of our method, called SimQ. We used the “AQUA” parser [18] to extract sentence syntactic structure. The UMLS [17] was used to annotate the sentences and generate semantic features. The Natural Language Parser (NLP) parsing results and semantic annotation were combined to create features for estimating similarity scores among question sentences.

###  Semantic Annotation and Medical Entity Recognition

Semantic annotation is a fundamental step of the proposed Q&A similarity analysis. The annotation procedure aims to identify health-related entities from the free-text consumer questions and assign semantic types to the identified entities. We performed named entity identification using an adapted semantic annotation tool that was developed from UMLS [19,20]. The annotation tool mapped the biomedical terms to the UMLS concepts and semantic types [17]. It has been demonstrated that UMLS-based lexicons cover a wide range of medical concepts [19-21] that can be annotated. Each of the extracted entities was assigned a Concept Unique Identifier (CUI) as defined in UMLS. Subsequently, we chunked the sentence into smaller segments based on the identified phrases and words [19]. For example, the question, “Could folic acid cause a bitter taste and body odor?” would be annotated and chunked as: “could// | folic acid/C0016410/Pharmacologic Substance | cause/C0678227/Functional Concept | a// | bitter taste/C0235290/Sign and Symptom | and// | body odor/C0085595/Finding”. Each chunk was separated by the “|” mark and consisted of three elements: the name entities (eg, folic acid, bitter taste), the concept identifiers (eg, C0016410, C0235290), and the semantic types (eg, Pharmacologic Substance, Sign and Symptom). Words without corresponding semantic mappings in UMLS were also kept to maintain sentence syntactic structure, such as the auxiliary verb “could” and the connector “and”. In this step, the identified name entities enhanced the following syntactic parsing. The annotated semantic types were then used for generating semantic features to analyze the question similarities of the consumer questions.

### Syntactic Features

**Figure 2:**
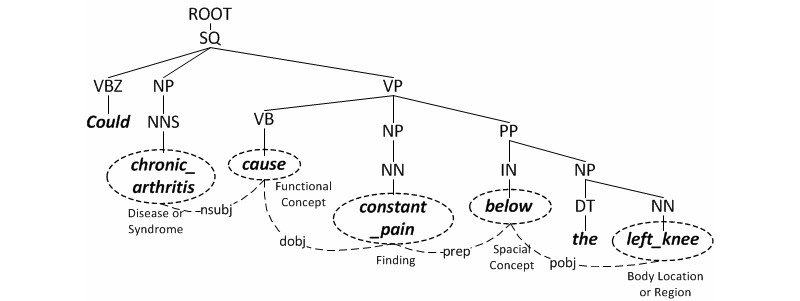
Parsed syntactic tree and semantic dependency.

To analyze the linguistic structure and the constituents of consumer questions, we parsed the question sentences into syntactic trees. The AQUA parser [18] was extended from the Stanford parser [22], and then used to construct syntactic trees and assign part-of-speech (POS) tagging. A parsed tree is a formalized structure that represents the syntactic relationship of the sentence constituents. For example, the syntactic tree in Figure 2 shows the parsing result of the sample sentence, “Could chronic arthritis cause constant pain below the left knee?”. The sentence root is tagged as a SQ (Simple Question). It is further parsed into three parts: VBZ (3^rd^ person verb), NP (noun phrase), and VP (verb phrase). The syntactic tree is expanded until all leaves contain a single constituent. Unlike standard syntactic parsing that treats each of the words as a constituent, our method uses the semantic annotation results and treats the UMLS recognizable entities as syntactic constituents. This last step enabled us to retrieve relationships between the identified named entities.

### Semantic Features

We constructed dependency grammars [22] from the syntactic tree, which represent grammatical relationships between the identified constituents. Studies have shown that dependency parsing can facilitate retrieving information from free-text within medical notes, such as from discharge summaries [18] or clinical research eligibility criteria [23]. A dependency grammar construct consists of a governor, a dependent, and a relationship name. In Figure 2, the governor and the dependent elements are encircled by dotted lines and linked together. For example, dependent “chronic arthritis” is a nominal subject (nsubj) of the governor “cause”, while “constant pain” is a direct object (dobj) of the governor “cause”. By applying the dependency relationship to the semantic annotation, we can extract semantic relationships between the entities. For example, we can extract the semantic relationship, “Disease or Syndrome (chronic arthritis) - Functional Concept (cause) - Finding (constant pain)”, which indicates that the disease has a functional influence on the clinical finding. Similarly, we can extract another relationship, “Finding (constant pain) - Spatial Concept (below) - Body Location or Region (left knee)”, which designates the spatial location of the clinical finding. The semantic-type pairs in the extracted semantic relationship were then used to represent semantic features for similarity calculation.

### Question Similarity

**Figure 3:**
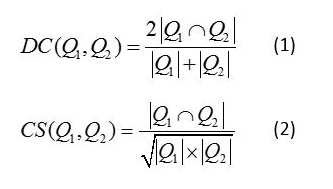
Dice coefficient (1) and cosine similarity (2) formulas.

Dice coefficient and cosine similarity are the algorithms that are employed for calculating similarity in this paper. Dice coefficient (DC) and cosine similarity (CS) (see Figure 3) were used to evaluate the similarity score between questions. The similarity score has a value range of 0-1. A score of zero means two questions are not similar at all, and a score of one means that they are completely the same. Assuming that there are two feature sets *Q*_1_ and *Q*_2_ that are generated from two different consumer questions, we can then calculate the DC and CS similarity scores through the formulas in Figure 3.

We use binary representation for both syntactic and semantic features. If a feature appears to a consumer in a question, then it has a value of 1; otherwise, the value is 0. From the binary representation, we can generate vectors containing both syntactic and semantic features to compare the similarity between these two questions as indicated in formulas 1 and 2 in Figure 3.

## Results

### Data Source

**Figure 4:**
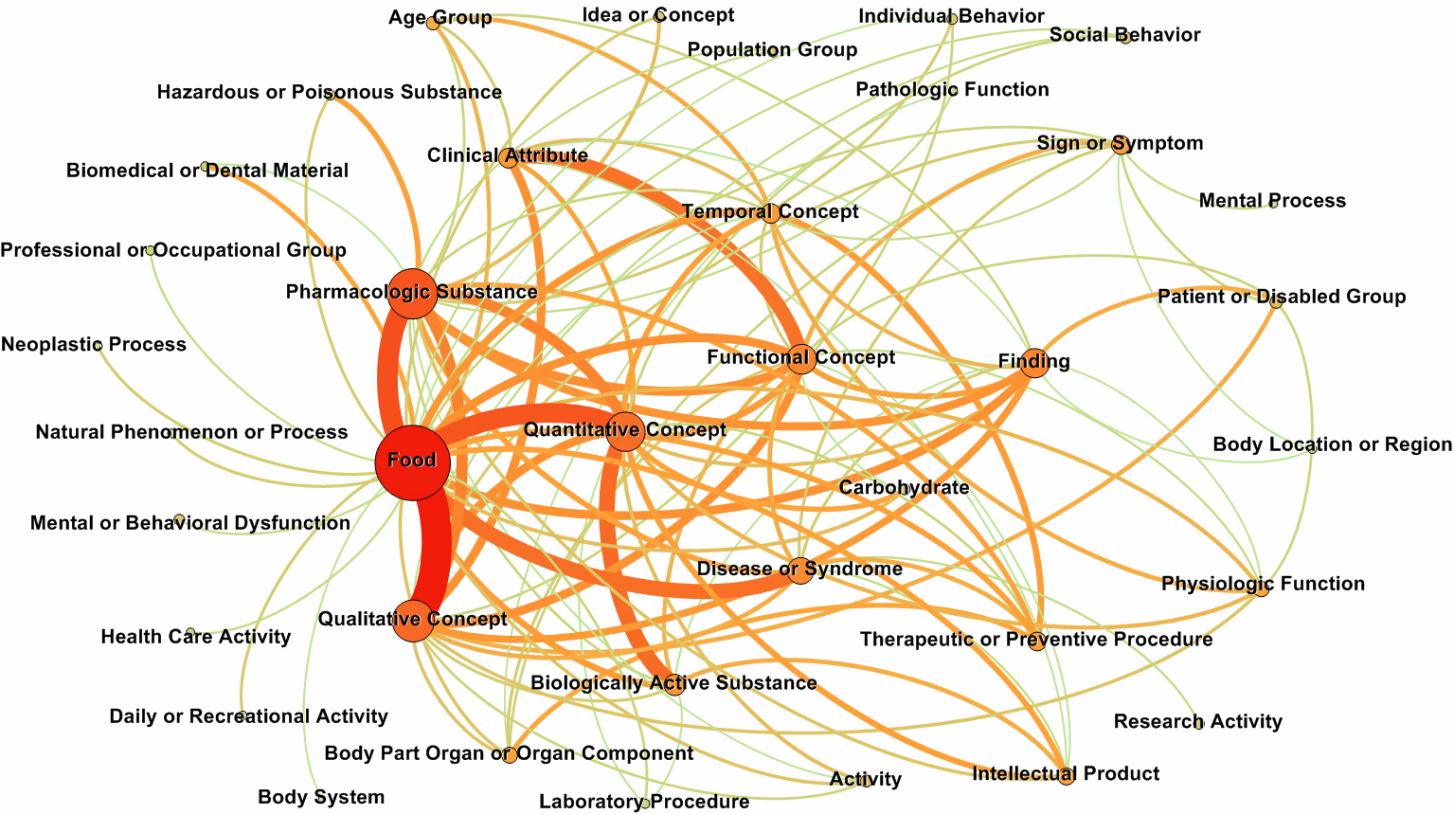
Overview of the semantic dependency network of the “Diet and Nutrition” topic.

We applied our method (SimQ) to the consumer questions posted on the Netwellness website, which has archived over 70,000 Q&As and more than 600 health information articles that were written by domain experts. All of the Q&As and articles were categorized into 121 health topics. The performance of the proposed SimQ method was then evaluated by using 2000 random selected NetWellness questions. The precision, recall, and *F*-score were measured. Also, we created an illustration for the aggregated semantic type patterns of the “Diet and Nutrition” category, which contained 2335 questions (see Figure 4).

### Semantic Dependency Overview

Figure 4 shows the overview of semantic type patterns in the topic group “Diet and Nutrition”. The nodes in the figure represent semantic types (eg, Food, Population Group, and Qualitative Concept, etc), while the edge that connects the two nodes indicates a dependency relationship between two types. The size of a node represents the frequency of the semantic types within the questions, and the width of an edge represents the frequency of the dependency relationships between two types. By connecting all semantic types (nodes) using their dependency relationships (edges), we were able to produce an overview of the semantic patterns. The result clearly shows the major topics and their connections in the “Diet and Nutrition” category. Among all 135 semantic types defined in UMLS, only 37 are used in this category. For example, in the semantic type, “Foods and medical substances”, quality and quantity attributes are the most prominent topics, such as dependency pairs “garlic − benefit”, “protein – amount”, and “grape seed extract − benefits and risks”. Diseases, symptoms, and medical findings associated with foods are also very popular questions, such as “gallstones − diet”, “heart disease − wine”, and “low blood sugar − food”. This result indicates that consumer questions within the same topic group share many similar patterns. We hypothesize that semantic features could be used further to improve similarity analysis.

### Example Results of Similar Questions

Table 1 shows some example results of similar questions retrieved from the NetWellness website. Given a particular consumer question, our algorithm will run through all of the archived questions on NetWellness to retrieve the top ranked similar questions. The top 5 similar questions and their similarity scores are shown in Table 1 using two examples of consumer health questions: “My throat glands feel swollen, help?” and “Low platelet counts”.

**Table 1:** Examples of SimQ calculated similar questions.

Rank	Similar questions		Similarity score
	**Input question: “My throat glands feel swollen, help?”**		
1		Swollen throat glands are sore?	0.7368
2		Sore throat and swollen glands?	0.6718
3		Swollen feeling in throat, can`t swallow well?	0.6545
4		My throat is sore all the time and also my glands?	0.5901
5		Painful swollen uvula, please help?	0.5611
	**Input question: “Low platelet counts?”**		
1		Less platelet count?	0.8235
2		What causes low platelet count?	0.7906
3		Extremely low platelet count?	0.7726
4		Decreased platelet count?	0.7003
5		Food for increase in platelet count?	0.5957

### Evaluation

To evaluate the performance of the SimQ method, 12 seed consumer questions were selected from Yahoo Answers as input questions. These questions were selected from different categories, such as women’s health, diseases and conditions, and mental health. Two biomedical informaticians, who were independent to this project, were recruited to generate a gold standard to evaluate the results of the SimQ question retrieval engine. They were asked to manually select Netwellness questions that were closely similar to the seed questions. A total of 2000 randomly selected Netwellness questions were used as the candidate pool. In total, 246 consumer questions were selected by the evaluators as the positive gold standard to evaluate SimQ’s retrieval performance. The initial agreement between the two evaluators was 83%. However, they were allowed to discuss and reach a final unanimous agreement on all the similar consumer questions, which were then used as the gold standard.

We also compared the performance of similarity analysis using different feature representations. Table 2 shows the results of SimQ using the Dice coefficient and cosine similarity algorithms. The baseline features (B) are the bag-of-words representation of a question, which is the standard representation of NLP analysis. The normalized features (N) are words that have been normalized by the Specialist Lexical Tool. The lexical tool normalizes plural terms and past tenses to their stem form. The concept features (C) are the UMLS concepts identified in the process of semantic annotation. The N+POS (P) features are the combination of normalized terms and their syntactic part-of-speech tagging. The N+Concept (NC) features are the combination of normalized terms and their mapped UMLS concepts. The N+C+Type (NCT) features are the combination of the precious feature (NC) and the extracted semantic type features described in the Semantic Features section.

In Table 2, we can see that Dice similarity performs better than cosine similarity in this task. The results indicate that word normalization, UMLS concepts, and semantic types improve similarity analysis. Part-of-speech tagging has no contribution to the similarity analysis. The best performing representation is the N+C+Type (NCT) features, with which the system achieved 75.0% *F*-score, 72.2% precision, and 78.0% recall.

Syntactic parsing is used to facilitate the identification of named entities and to support the construction of semantic features [24]. Part-of-speech tagging was evaluated as a syntactic feature. The evaluation result shows that part-of-speech did not improve the performance of retrieval. The semantic features are constructed from the semantic type pairs, which have been extracted from the parsed dependency tree. The evaluation result shown indicates that the semantic features improved the retrieval results, while syntactic parsing had little effect. From our analysis, the contributions of semantic features work in two aspects: (1) the semantic features strengthen key medical concepts and reduce the weight of non-medical concepts, and (2) the semantic features improve semantic similarity analysis of consumer questions that cannot be ascertained directly from the text. For example, questions such as “Could my blurred eyes caused by hypertension?” and “HBP lead to blurry vision?” share the same semantic concepts, Blurred Vision (CID:C0344232) Hypertensive Disease (CID:C0020538), and the same semantic type pattern, Disease_or_Syndrome - cause - Sign_or_Symptom. Figure 4 shows that there are many overlapping semantic relationships (semantic dependency pairs) within a closely related topic group.

**Table 2:** Evaluation of different feature representations for consumer Q&A similarity analysis (average of 12 experiments using 12 seed questions).

Feature		True positive	False positive	True negative	False negative	Precision %	Recall %	*F*-score %
**Algorithm - Cosine Similarity**								
	Baseline (B)	12.83	7.67	1969.33	7.67	62.6%	62.6%	62.6%
	Normalized (N)	12.67	6.67	1970.33	7.83	65.5%	61.8%	63.6%
	Concept (C)	14.00	8.33	1968.67	6.50	62.7%	68.3%	65.4%
	N+POS (P)	11.67	7.67	1969.33	8.83	60.3%	56.9%	58.6%
	N+ Concept (NC)	15.00	7.50	1969.50	5.50	66.7%	73.2%	69.8%
	N+C+Type (NCT)	15.33	6.67	1970.33	5.17	69.7%	74.8%	72.1%
**Algorithm - Dice Similarity**								
	Baseline (B)	11.33	3.17	1973.83	9.17	78.1%	55.3%	64.7%
	Normalized (N)	15.50	10.33	1966.67	5.00	60.0%	75.6%	66.9%
	Concept (C)	15.33	8.00	1969.00	5.17	65.7%	74.8%	70.0%
	N+POS (P)	11.67	5.67	1971.33	8.83	67.3%	56.9%	61.7%
	N+ Concept (NC)	14.33	3.83	1973.17	6.17	78.9%	69.9%	74.1%
	N+C+Type (NCT)	16.00	6.17	1970.83	4.50	72.2%	78.0%	75.0%

### SimQ Application

**Figure 5:**
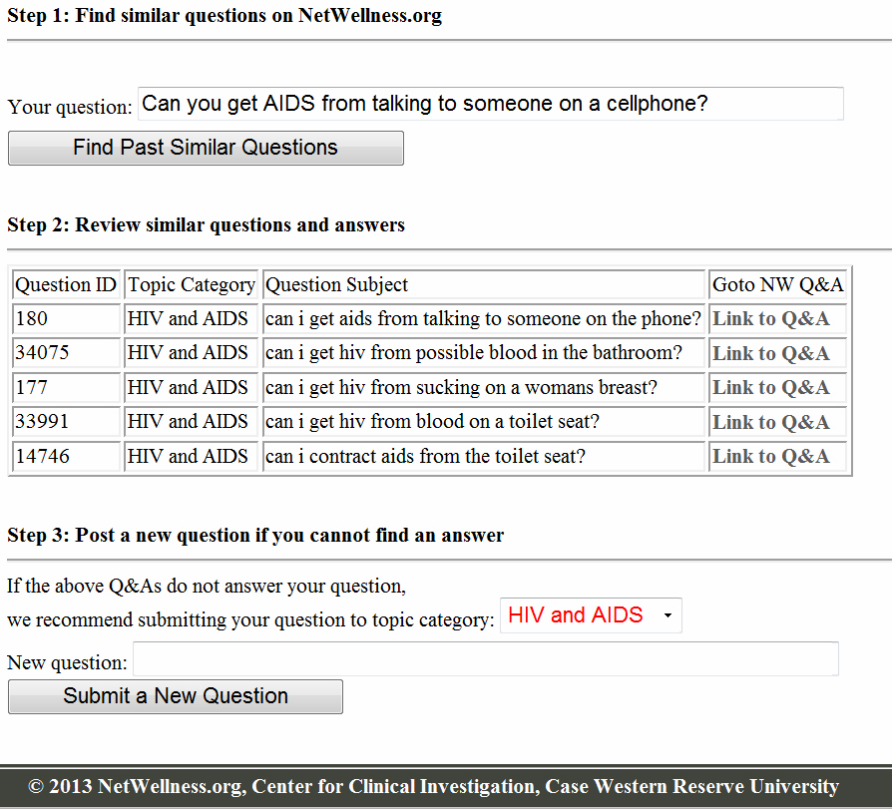
Application of SimQ for NetWellness.

To demonstrate the use of SimQ, we developed an application that complemented the existing Q&A services on the NetWellness website. Figure 5 shows the Web interface of SimQ. The original Q&A service on NetWellness prompts users to select a topic category among 120 categories, and subsequently allows consumers to submit their question to a specific category. A coordinator reviews the question and then determines whether the user-assigned category is correct. If the question is submitted to the correct category, the coordinator will forward the question to a health expert.

This new application enhances the workflow through semantic similarity analysis (see Figure 5, Step 1). Consumers first submit their health concerns to the SimQ question retrieval engine. SimQ analyzes the question and calculates the similarities against all the questions that have been archived on Netwellness (Figure 5, Step 2). A list of closely related similar questions will be retrieved for the consumer. The user can then browse through similar questions that were posted in the past and read the related Q&As. This step improves the consumer’s understanding of the health problem using historical knowledge. The consumer could also find the right answer for their problem directly from the archived Q&As. After the consumers have read through the similar Q&As, and have determined that they wish to continue submitting a new question, the system will automatically recommend one or more topic categories for them to consider using the most frequent topics that have been assigned in the past applying similar questions (Figure 5, Step 3). This important step addresses the problem of wrong category selection, which is very commonly encountered on public Web-based Q&A services. Wrong category submission may lead to no answer or even require manual correction. The application described above demonstrates that our method can be integrated into existing systems to improve the service quality of the Q&A workflow.

## Discussion

### Q&A Retrieval

At present, one of the most common ways to find health information online is through search engines. Search engines use keyword-based information retrieval techniques [25], which return ranked Web pages that contain the searched keyword. While returning top-ranked documents can be useful in some cases, frequently this type of search does not satisfy the user’s information needs [26], as has been discussed in TREC, an international information retrieval consortium. Hence, despite the common use of search engines, community-based Q&A platforms are becoming more popular because they address the fundamental need to get human (consumer or expert) answers for health questions [7,27]. NetWellness is a not-for-profit platform that provides expert-answered recommendations to consumers for solving their health questions, which provides an invaluable resource for consumers.

### Related Prior Work

The SimQ method is related to but fundamentally different than Question Answering Machines (QAMs) [16,26]. QAMs aim to automatically answer human questions by using computer programs supported by artificial intelligence techniques [28]. There are various types of QAMs in existence today. In the biomedical information domain, AskHERMES [29] is an automated system that finds and filters clinical information to help physicians obtain relevant information. Patrick and Li [30] developed an ontology to classify questions from intensive care units. MiPACQ [31,32] is a system that integrates different data sources to answer clinical questions. MEDLINE is the largest QAM database, and contains 20 million references to PubMed articles. Sneiderman et al [33] evaluated the performance of three methods in answering clinical queries using MEDLINE and found that external semantic knowledge improved the performance of two of these methods. Automatic machine question answering is still a very challenging task, especially for health informatics applications. Most of the machine answering systems can only provide factual answers to the questions. For tasks that involve questions about advice and/or opinions for consumer health problems, especially when the question is presented in a free-text format, the performance of these systems is still not satisfactory [34]. For example, one would need sophisticated reasoning ability and professional pharmaceutical knowledge to answer the question: “Why Fosamax should not be taken with estrogen?” SimQ is fundamentally different, since it reuses similar questions from the archived knowledge base to satisfy consumers’ information needs to complement the existing research of machine answering systems. Therefore, our study is specifically focused on improving the information retrieval of community-based Q&A services instead of QAMs. The SimQ method analyzes question-to-question similarities in the archived Q&A database and retrieves relevant Q&As to address consumers’ health concern. As far as we know, this is the first research study with a primary focus on analyzing the similarity of consumer health questions.

### Error Analysis

We observed two types of errors from the SimQ retrieval results: false positive results and false negative results. False positive results, which are incorrectly included questions, were often created by questions with small but important differences. For example, SimQ retrieves the query, “How can I lose weight in one month?” for the question, “How I can gain weight quickly”, because both “lose weight” and “gain weight” have the same semantic type, Findings. Both questions contain the same semantic types, Patient Group and Temporal Concept. The only major difference is seen in the concepts of “lose weight (CUI:C0043096)” and “gain weight (CUI:C0043094)”. One potential solution for this type of error is to incorporate concept importance ranking into the similarity analysis. When generating feature vectors, important concepts have higher weights for calculating the similarity score, which can improve the retrieval results. False negative results (incorrectly excluded questions) are often caused by complex questions. For example, the question “I have a breast lump, could it be a lymph node or tumor?” is semantically close to “Is swelling breast a sign of breast cancer?” by human standards. However, the SimQ similarity score is not very high. To address this problem, we need to add concept reasoning ability to the similarity analysis. In this example, the concept “breast lump (CUI:C0424849)” is a descendent of the concept “swelling (CUI:C0038999)”, and “breast cancer (CUI:C0006142)” is a descendent of “tumor (CUI:C0027651)”.

Short ambiguous questions can also lead to both false negative and false positive errors. For example, when analyzing the question, “Vitamin B6 deficiency”, SimQ retrieved the false positive result “Vitamin B12 deficiency?” and the false negative result “What are the symptoms of Vitamin B6 deficiency?” We believe that potential methods to address errors created by short ambiguous questions include weighting the question elements by importance and/or applying a query expansion technique. For example, an intuitive way to expand the question for Netwellness is to include previous answers from similarity analysis. However, the answers usually are much more complex and longer than the question, so it is still challenging to achieve a good result, especially since real-time retrieval response is needed. Integrating both questions and answers to improve retrieval results will be examined in subsequent studies that we plan to undertake.

### Limitations

SimQ uses UMLS as a standardized semantic knowledge source. In the future, we plan to exploit other medical knowledge sources for semantic annotation, which could provide finer granularity of the semantic assignment and improve semantic analysis. Additionally, some researchers have pointed out that Consumer Health Vocabularies (CHV) [35] may facilitate natural language processing of consumer-related free text. Because most questions submitted to NetWellness are consumer questions about health, a natural extension to our current approach will be to evaluate the effectiveness of consumer health vocabularies in future studies.

### Conclusions

Similarity analysis of consumer health questions can significantly improve the quality and accessibility of online community-based question answering (Q&A) services. In this study, we proposed a new application called SimQ, which analyzes the semantic similarity of consumer health questions by combining natural language processing and semantic pattern techniques. The evaluation results show that our approach effectively retrieves similar questions on NetWellness. The results show that SimQ reached the highest precision of 72.2%, recall of 78.0%, and *F*-score of 75.0%. We demonstrated a use case application by designing a new Q&A pipeline for the NetWellness website, which retrieves previous Q&As similar to the user’s health care. We designed a new Q&A pipeline for NetWellness, which retrieves previous Q&As similar to the user’s health care question. Then we demonstrated by using a particular case how the additional features of SimQ would be applied to a health consumer’s inquiry and integrated into the existing system to improve the service quality of the Q&A workflow. Therefore, we have shown that SimQ not only reduces response delay by instantly providing closely related question and answers, but also helps consumers improve the understanding of their health concerns.

## Multimedia Appendix 1

Source code for similarity analysis.

## Abbreviations

CTSC: Clinical and Translational Science Collaborative Cleveland
NCATS: National Center for Advancing Translational Science
NLP: natural language processing
NP: noun phrase
POS: part-of-speech
Q&A: question-and-answer
SQ: Simple Question
UMLS: Unified Medical Language System
VB: verb phrase
VBZ: 3rd person verb
